# An Unusual Etiology of Cauda Equina Syndrome in an AIDS Patient: A Case Report

**DOI:** 10.7759/cureus.107634

**Published:** 2026-04-24

**Authors:** Wesley A Kim, Daniel Dominguez, Adiam Yonas

**Affiliations:** 1 Diagnostic Radiology, Rowan-Virtua School of Osteopathic Medicine, Stratford, USA; 2 Internal Medicine, Hoboken University Medical Center, Hoboken, USA

**Keywords:** aids, cauda equina syndrome, drop metastases, ebv, hiv, immunosuppression, leptomeningeal spread, myelopathy, spinal cord compression, systemic diffuse large b-cell lymphoma

## Abstract

Patients with advanced HIV/AIDS are at high risk for Primary central nervous system lymphoma (PCNSL) due to impaired immune surveillance and HIV-driven B-cell proliferation. While intracranial lesions are well recognized, spinal involvement remains underrecognized due to the scarcity of well-documented reported cases in the current literature.

In this report, we describe the case of a 35-year-old male with a new HIV diagnosis who experienced acute bilateral lower extremity weakness and urinary retention during hospitalization for fever, perianal abscess, and urinary tract infection. Initial CD4 count revealed profound immunosuppression, with a CD4 count of less than 20 cells/μL and a viral load of 178,000 copies/mL. Spinal MRI demonstrated an enhancing mass at T2-T3, with surrounding edema extending to C5-C6 and multiple vertebral lesions, while brain MRI was unremarkable. Liver biopsy confirmed Epstein-Barr virus (EBV)-positive diffuse large B-cell lymphoma (DLBCL), establishing a diagnosis of AIDS-associated systemic lymphoma with secondary central nervous system (CNS) involvement presenting as cauda equina syndrome - a neurosurgical emergency with potential for irreversible paralysis.

This case underscores the importance of early, comprehensive spinal imaging in HIV-positive patients with new-onset myelopathy or cauda equina symptoms.

## Introduction

Primary central nervous system lymphoma (PCNSL) is a distinct subtype of extranodal non-Hodgkin lymphoma that arises exclusively within the nervous system, including the brain, spinal cord, leptomeninges, and eyes, without evidence of systemic disease [1-6]. However, systemic diffuse large B-cell lymphoma (DLBCL) can also involve the central nervous system (CNS) secondarily, and distinguishing between these two entities is critical, as the diagnosis of PCNSL requires the absence of lymphomatous disease outside the CNS [2]. When systemic involvement is identified, the case is reclassified as secondary CNS lymphoma, which carries distinct staging, treatment, and prognostic implications [2]. In HIV-positive patients with advanced immunosuppression, both PCNSL and systemic DLBCL with secondary CNS involvement must be considered in the differential diagnosis of new CNS lesions [1,2,7].

In patients with HIV/AIDS, PCNSL develops due to impaired immune surveillance across the blood-brain barrier combined with Epstein-Barr virus (EBV)-driven B-cell proliferation [1,2,7]. Tumorigenesis involves dysregulation of proto-oncogenes such as MYC, PIM1, RHOH, and PAX5, alongside monoclonal EBV genomic integration [2]. The majority of these neoplasms are classified as high-grade B-cell tumors that demonstrate germinal center marker expression (BCL-6) and pan-B-cell antigen positivity for CD19, CD20, CD22, and CD79a [2].

Before the availability of combination antiretroviral therapy (ART), HIV-positive individuals carried a 100- to 500-fold increased likelihood of developing non-Hodgkin lymphoma relative to the uninfected population [1]. Since ART became widely available, the incidence of HIV-associated CNS lymphoma has substantially declined, though it remains a significant cause of morbidity in patients with advanced immunosuppression [2]. Of the B-cell non-Hodgkin lymphoma subtypes occurring in HIV, CNS involvement correlates most strongly with severe immune depletion and typically manifests at the nadir of CD4 cell counts [1]. Secondary CNS involvement by systemic DLBCL occurs in approximately 5% of cases and carries a particularly poor prognosis [2].

Although intracranial CNS lymphoma is well characterized, spinal cord involvement remains exceptionally rare and underreported, regardless of whether the disease is primary or secondary [2,3]. This underrecognition poses significant clinical danger, as spinal cord compression and cauda equina syndrome represent neurosurgical emergencies where delayed diagnosis leads to irreversible neurological deficits and possible death.

## Case presentation

Initial presentation

A 35-year-old male arrived at the emergency department reporting three days of progressively worsening right gluteal pain and perianal swelling, accompanied by subjective fevers, chills, and discomfort while seated. He denied rectal bleeding. Additionally, he noted dysuria and increased urinary frequency. The patient reported unprotected sex with multiple male partners and had no prior HIV testing. He had no established primary care and had not undergone sexually transmitted infection screening or hepatitis A/B vaccination.

Past medical history was unremarkable. Past surgical history included appendectomy. He had no known drug allergies. Social history was notable for the patient identifying as a male who is sexually active with male partners, occasional alcohol consumption (approximately one to two drinks per week), and no reported tobacco or illicit drug use.

Emergency department course

On initial evaluation, vital signs were a temperature of 100.4°F, heart rate of 112 beats per minute, blood pressure of 118/72 mmHg, respiratory rate of 18 breaths per minute, and oxygen saturation of 97% on room air. Physical examination revealed an alert but uncomfortable-appearing male. Rectal examination demonstrated a small, tender, fluctuant mass at the seven o’clock position, with surrounding erythema but no tracking or crepitus. Neurological examination at presentation was grossly intact, with full strength in all extremities and no sensory deficits.

Laboratory studies revealed a white blood cell count of 4.5 K/μL (reference range 3.6-10.2 K/μL), hemoglobin of 9.8 g/dL with macrocytosis (mean corpuscular volume 108 fL), and a normal creatinine of 0.9 mg/dL. Serum albumin was low at 2.6 g/dL, and aspartate aminotransferase was mildly elevated at 48 U/L. Lactate dehydrogenase was markedly elevated at 420 U/L. Inflammatory markers were significantly elevated, with an erythrocyte sedimentation rate of 65 mm/hr (reference range 0-22 mm/hr) and C-reactive protein of 45.2 mg/L (reference range <10.0 mg/L). Urinalysis was positive for leukocyte esterase, nitrites, greater than 50 white blood cells, and bacteriuria. HIV-1 screening by rapid test and enzyme immunoassay returned positive; the HIV-1 and HIV-2 antigen/antibody fourth-generation test was reactive. Confirmatory testing, including CD4 count and viral load, was sent. Serial laboratory values, vital signs, and microbiological results throughout the hospitalization are presented in Table 1.

**Table 1:** Serial laboratory values, vital signs, and microbiological results during hospitalization * Denotes abnormal value; (-) = Not obtained on that day; Day 1 = ED admission (06/06/2025). Additional serologies not shown: FTA-ABS reactive (06/13/2025); CMV DNA 207 IU/mL (07/01/2025); QuantiFERON-TB indeterminate; hepatitis B surface antibody positive, surface antigen negative; hepatitis C antibody nonreactive. Abbreviations: WBC, white blood cell count; MCV, mean corpuscular volume; BUN, blood urea nitrogen; AST, aspartate aminotransferase; ALT, alanine aminotransferase; ALP, alkaline phosphatase; LDH, lactate dehydrogenase; CD4, cluster of differentiation 4; ESR, erythrocyte sedimentation rate; CRP, C-reactive protein; EBV, Epstein-Barr virus; PCR, polymerase chain reaction; HSV, herpes simplex virus; IgG, immunoglobulin G; Ag, antigen; RPR, rapid plasma reagin; HPF, high-power field; ESBL, extended-spectrum beta-lactamase; SpO_2_, peripheral oxygen saturation; ED, emergency department; FTA-ABS, fluorescent treponemal antibody absorption; CMV, cytomegalovirus; TB, tuberculosis.

Laboratory Parameter	Reference Range	Day 1 (06/06)	Day 2 (06/07)	Day 3 (06/08)	Day 4 (06/09)	Day 5 (06/10)	Day 6 (06/11)	Day 8 (06/13)
WBC (K/uL)	3.6-10.2	4.5	5.4	2.2*	1.9*	2.0*	2.5*	1.8*
Hemoglobin (g/dL)	13.5-17.5	9.8*	9.5*	9.2*	8.9*	8.7*	8.8*	8.6*
Hematocrit (%)	38.3-48.6	30.2*	29.4*	28.5*	27.6*	27.0*	27.2*	26.8*
MCV (fL)	80-100	108*	109*	110*	108*	109*	110*	111*
Platelets (K/uL)	150-400	165	158	142*	135*	128*	130*	122*
Sodium (mEq/L)	136-145	137	136	135*	134*	135*	136	135*
Potassium (mEq/L)	3.5-5.0	4.0	3.8	3.7	3.6	3.8	3.9	3.7
Chloride (mEq/L)	98-106	101	100	99	98	99	100	99
BUN (mg/dL)	7-20	16	18	22*	24*	21*	19	17
Creatinine (mg/dL)	0.7-1.3	0.9	0.9	1.0	1.1	1.0	0.9	0.9
Glucose (mg/dL)	70-100	105*	98	94	92	90	95	88
Albumin (g/dL)	3.5-5.5	2.6*	2.5*	2.4*	2.3*	2.3*	2.4*	2.3*
AST (U/L)	10-40	48*	52*	50*	46*	44*	42*	40
ALT (U/L)	7-56	42	45	48	46	44	40	38
ALP (U/L)	44-147	132	138	145	152*	148*	142	140
LDH (U/L)	140-280	420*	-	445*	-	430*	-	405*
CD4 Count (cells/uL)	500-1500	-	<20*	-	-	-	-	-
CD4 (%)	30-60%	-	1%*	-	-	-	-	-
HIV-1 Viral Load (copies/mL)	Not detected	-	178,000*	-	-	-	-	-
ESR (mm/hr)	0-22	65*	-	72*	-	68*	-	60*
CRP (mg/L)	<10.0	45.2*	-	50.8*	-	42.5*	-	36.1*
EBV DNA PCR (IU/mL)	Not detected	-	-	-	-	2,300*	-	-
Beta-(1,3)-D-Glucan (pg/mL)	<60	-	-	112*	-	-	-	-
HSV-1 IgG (index)	<0.90	27.10*	-	-	-	-	-	-
HSV-2 IgG (index)	<0.90	3.25*	-	-	-	-	-	-
Cryptococcal Ag	Negative	-	-	-	-	Negative	-	-
RPR	Nonreactive	Nonreactive	-	-	-	-	-	-
Leukocyte Esterase	Negative	Positive*	-	-	-	-	-	-
Nitrites	Negative	Positive*	-	-	-	-	-	-
WBC/HPF	0-5	>50*	-	-	-	-	-	-
Urine Bacteria	None	Many*	-	-	-	-	-	-
Blood Culture (1 of 2)	No growth	-	-	-	*Salmonella* spp.*	-	-	-
Wound Culture (Perianal)	No growth	-	-	ESBL E. coli*	-	-	-	-
Temperature (F)	97.8-99.1	100.4*	99.8*	99.2*	98.8	98.6	98.4	98.6
Heart Rate (bpm)	60-100	112*	108*	98	92	88	85	82
Blood Pressure (mmHg)	90/60-120/80	118/72	115/70	110/68	108/65	105/62	92/55*	107/67
Respiratory Rate	12-20	18	18	16	16	18	18	16
SpO_2_ (%)	95-100	97	98	98	97	98	98	100

CT of the abdomen and pelvis revealed a perianal fluid collection without deep extension. Incision and drainage of the perianal abscess were performed in the emergency department. The patient was started on trimethoprim-sulfamethoxazole 800 mg-160 mg by mouth daily for abscess coverage and concurrent urinary tract infection. Blood and urine cultures were obtained. The patient was admitted for HIV workup, urinary tract infection treatment, and abscess management. Infectious disease, gastroenterology, and surgery consultations were placed.

Hospital course - initial improvement (hospital days 2-3)

On hospital day 2, the patient reported mild improvement in right buttock pain and perianal swelling following incision and drainage. Vital signs trended toward improvement, with temperature decreasing to 99.8°F and heart rate to 108 beats per minute. CD4 count and viral load results returned, revealing profound immunosuppression, with a CD4 count of less than 20 cells/μL (1% of total lymphocytes) and an HIV-1 viral load of 178,000 copies/mL. Confirmatory HIV testing was completed, and infectious disease consultation was in progress.

By hospital day 3, wound culture from the perianal abscess grew extended-spectrum beta-lactamase (ESBL)-producing *Escherichia coli*. Additional serologies demonstrated a reactive fluorescent treponemal antibody absorption (FTA-ABS) test with a nonreactive rapid plasma reagin (RPR), consistent with previously treated or late latent syphilis. Herpes simplex virus (HSV)-1 IgG was positive at 27.10 index (reference <0.90), and HSV-2 IgG was positive at 3.25 index, indicating prior HSV-1 and HSV-2 exposure. Beta-(1,3)-D-glucan was elevated at 112 pg/mL (reference <60 pg/mL), raising concern for invasive fungal infection. EBV DNA polymerase chain reaction returned at 2,300 IU/mL, and cytomegalovirus (CMV) DNA quantitative polymerase chain reaction was subsequently found to be 207 IU/mL. Cryptococcal antigen was negative. QuantiFERON-TB Gold testing was indeterminate, likely secondary to profound immunosuppression. Antibiotics were continued, and warm compresses and sitz baths were maintained for the perianal abscess.

On hospital day 4, one of two sets of blood cultures grew *Salmonella* species, consistent with nontyphoidal *Salmonella* bacteremia - a recognized AIDS-defining illness in the setting of recurrent episodes [8,9]. Antibiotic coverage was broadened to ceftriaxone 2 g intravenously daily.

Clinical deterioration (hospital days 5-8)

During the hospitalization, the patient developed acute bilateral lower extremity weakness, saddle anesthesia, and urinary retention, prompting urgent evaluation for cauda equina syndrome. Neurological examination at the time of deterioration revealed bilateral lower extremity weakness with decreased motor strength (3/5 hip flexion, 3/5 knee extension, and 4/5 ankle dorsiflexion bilaterally), diminished sensation in the perianal region (saddle anesthesia), bilaterally positive Babinski signs, and absent bulbocavernosus reflex. Rectal tone was diminished. A Foley catheter was placed for urinary retention, with an initial residual volume of approximately 600 mL. The presence of bilaterally positive Babinski signs indicated upper motor neuron involvement attributable to the T2-T3 intramedullary mass, while the lower motor neuron findings - saddle anesthesia, urinary retention, and absent bulbocavernosus reflex - were consistent with involvement at the level of the conus medullaris and cauda equina by the distal vertebral lesions. This mixed presentation reflected the multifocal nature of the spinal disease. A blood pressure of 92/55 mmHg was noted on hospital day 6, consistent with the patient's chronically low baseline throughout the admission. No acute hemodynamic intervention was necessary.

This degree of immunosuppression placed the patient at the highest risk for opportunistic infections and AIDS-defining malignancies, particularly primary CNS lymphoma [1,4,7]. Additional workup revealed left lower lobe pneumonia and multiple hepatic lesions on cross-sectional imaging, raising concern for disseminated opportunistic infection versus malignancy.

**Figure 1:**
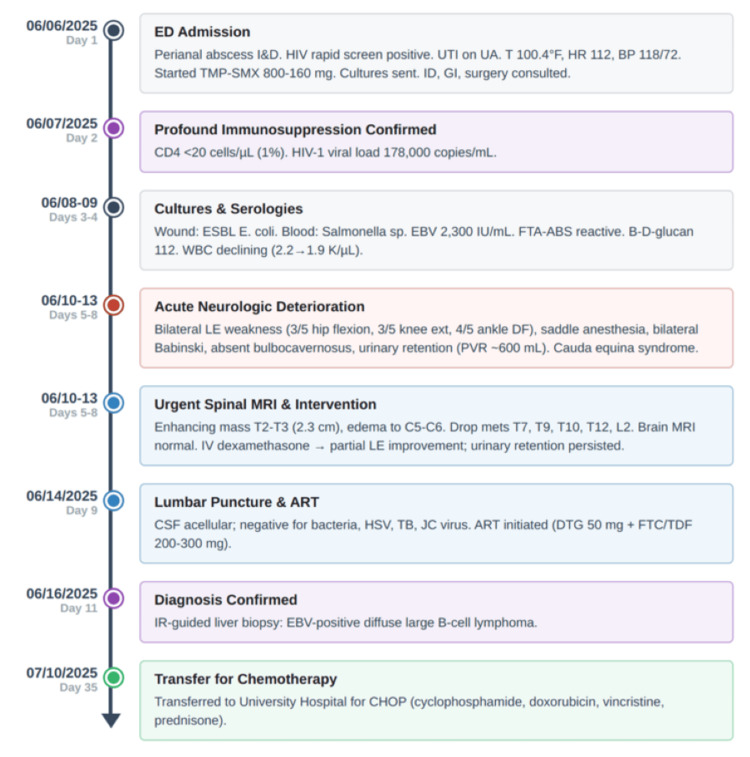
Timeline depicts key clinical events from emergency department (ED) admission through transfer for chemotherapy Abbreviations: ED, emergency department; I&D, incision and drainage; HIV, human immunodeficiency virus; UTI, urinary tract infection; UA, urinalysis; T, temperature; HR, heart rate; BP, blood pressure; TMP-SMX, trimethoprim-sulfamethoxazole; ID, infectious disease; GI, gastroenterology; CD4, cluster of differentiation 4; ESBL, extended-spectrum beta-lactamase; EBV, Epstein-Barr virus; FTA-ABS, fluorescent treponemal antibody absorption; WBC, white blood cell count; LE, lower extremity; DF, dorsiflexion; MRI, magnetic resonance imaging; IV, intravenous; CSF, cerebrospinal fluid; HSV, herpes simplex virus; TB, tuberculosis; ART, antiretroviral therapy; DTG, dolutegravir; FTC/TDF, emtricitabine/tenofovir disoproxil fumarate; IR, interventional radiology; CHOP, cyclophosphamide, doxorubicin, vincristine, and prednisone; PVR, post-void residual.

The clinical timeline from initial presentation through diagnosis and treatment initiation is summarized in Figure 1.

Neuroimaging

Given the acute myelopathy and concern for cauda equina syndrome, an urgent spinal MRI was obtained. Imaging demonstrated an expansile enhancing intramedullary lesion at T2-T3 extending approximately 2.3 cm in longitudinal dimension, encompassing nearly the entire cord at that level. There was an increased T2 signal extending superiorly and inferiorly to this lesion, with edema reaching rostrally to C5-C6. Multiple vertebral body lesions were identified at T7, T9, T10, T12, and L2, consistent with osseous metastatic involvement. At T10-T11, there was a shallow left posterolateral/foraminal protrusion with mild left lateral recess and neuroforaminal stenosis. At T11-T12, a shallow right posterolateral protrusion was noted without significant stenosis. Brain MRI was unremarkable, with no intracranial mass lesions identified.

The imaging findings were characteristic of spinal cord lymphoma. Primary intramedullary spinal cord lymphoma typically demonstrates multifocal, persistently enhancing lesions on spinal MRI, with involvement of the conus medullaris, cauda equina, or both [3]. The unremarkable brain MRI in this patient demonstrates that spinal PCNSL may develop independently of intracranial disease; in rare instances, the disease may be confined to the leptomeningeal compartment or involve the spinal cord in isolation [2].

**Figure 2:**
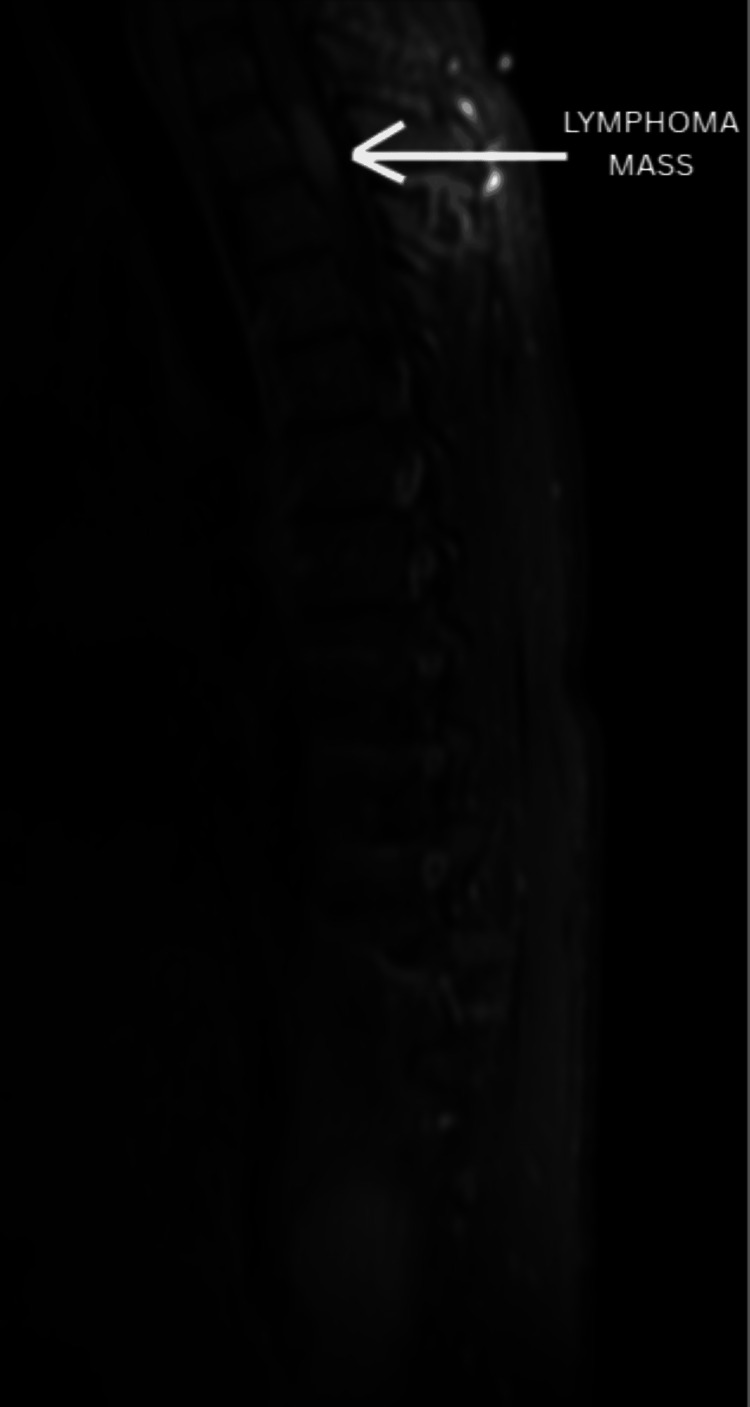
Sagittal T2-weighted MRI Sagittal T2-weighted MRI demonstrates an expansile intramedullary lesion centered at T2-T3 (arrow), in the proximal thoracic cord, measuring approximately 2.3 cm in length and involving nearly the entire cord at this level. Associated T2 hyperintensity extends above and below the lesion. Abbreviation: MRI, magnetic resonance imaging.

**Figure 3:**
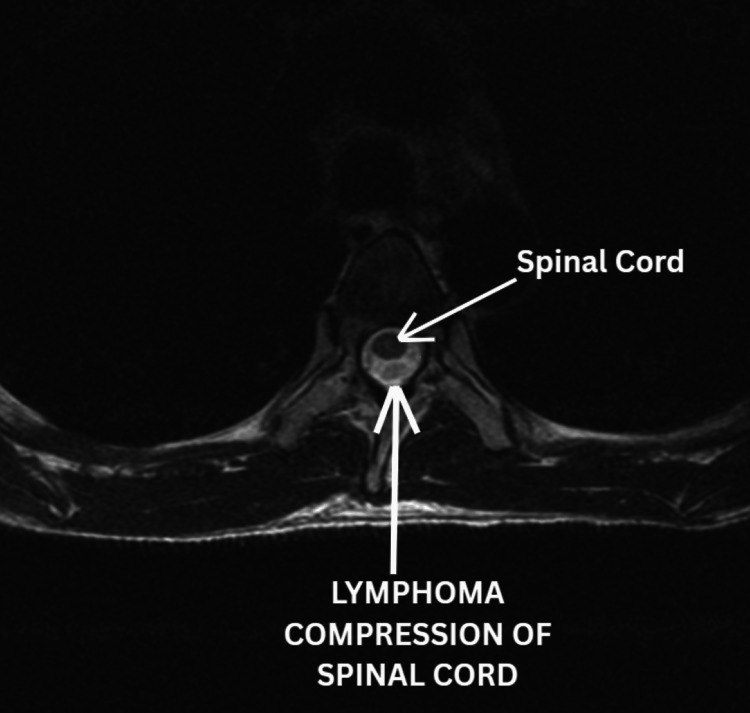
Axial T2-weighted MRI of the thoracic spine Axial T2-weighted MRI of the thoracic spine at the T2-T3 level demonstrates an expansile intramedullary lesion (arrow) with compression of the spinal cord by lymphoma. Abbreviation: MRI, magnetic resonance imaging.

**Figure 4:**
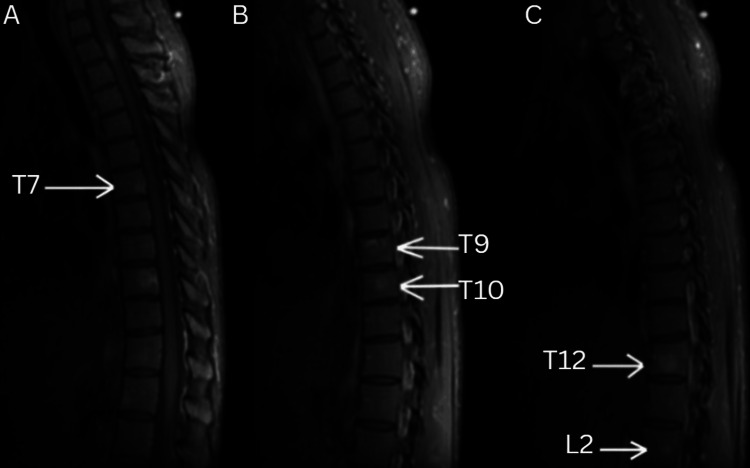
Sagittal T1 non-contrast MRI (A) Sagittal T1 non-contrast MRI demonstrating metastatic lesions within the T7 vertebral body (arrow). (B) Sagittal T1 non-contrast MRI showing additional vertebral body lesions at T9 and T10 (arrows). (C) Sagittal T1 non-contrast MRI of the thoracolumbar junction demonstrating metastatic lesions within the T12 and L2 vertebral bodies (arrows). Abbreviation: MRI, magnetic resonance imaging.

The following imaging was obtained during the diagnostic workup. A representative sagittal MRI of the thoracic spine demonstrating the T2-T3 lymphoma mass is shown in Figure 2. An axial view at the T2-T3 level further illustrates the expansile intramedullary lesion with associated spinal cord compression in Figure 3. Multiple vertebral body lesions were identified at T7, T9, T10, T12, and L2, consistent with osseous metastatic disease, as shown in Figure 4.

Mechanisms of spinal metastatic spread in lymphoma

Spinal metastatic spread in lymphoma occurs through two principal mechanisms. Hematogenous dissemination results in osseous deposits within the vertebral bodies, as observed in this patient at T7, T9, T10, T12, and L2. Alternatively, malignant lymphoma cells may disseminate through the cerebrospinal fluid (CSF) and seed distant sites along the spinal cord and nerve roots, a process termed leptomeningeal or drop metastases [9]. Lymphoma cells shed into the subarachnoid space are carried by CSF flow to deposit at lower spinal levels, most commonly in the thoracolumbar region [9]. Lymphoma has a natural tropism for the leptomeninges, with 5%-10% of systemic lymphomas spreading to the leptomeninges and 10%-16% of spinal epidural lymphomas developing subarachnoid spread [8,10,11].

**Figure 5:**
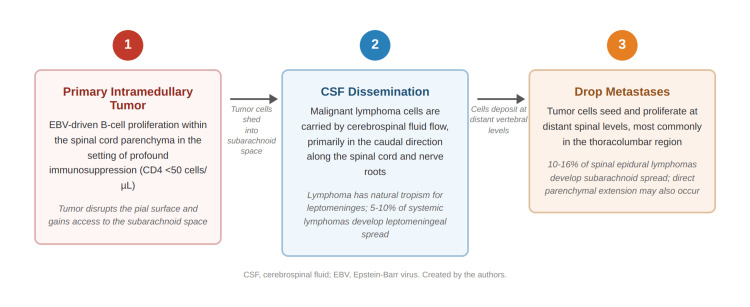
Mechanism of leptomeningeal spread and drop metastases in spinal lymphoma Schematic illustrating the pathway of CSF dissemination of malignant lymphoma cells from a primary intramedullary lesion to distant spinal levels. Source: Created by the authors based on published literature [8-11]. Abbreviations: CSF, cerebrospinal fluid; EBV, Epstein-Barr virus.

The mechanism of leptomeningeal spread and CSF-mediated drop metastases is illustrated in Figure 5.

Diagnosis and management

Cauda equina syndrome secondary to spinal CNS lymphoma was identified as a neurosurgical emergency, given the risk of irreversible neurologic loss. Neurosurgery was emergently consulted for consideration of surgical decompression and tissue diagnosis. The neurosurgical team recommended against operative intervention given the intramedullary location of the lesion and the high risk of worsening neurological deficits with surgical manipulation. Medical management with intravenous corticosteroids was favored, with plans to pursue a less invasive approach for tissue diagnosis.

Intravenous dexamethasone 10 mg every six hours was initiated on hospital day 5 to reduce peritumoral edema and cord compression. Following dexamethasone administration (hospital days 5-8), the patient demonstrated partial improvement in bilateral lower extremity strength, with hip flexion improving from 3/5 to 4/5 and knee extension from 3/5 to 4/5 bilaterally. However, urinary retention persisted.

Lumbar puncture was performed on hospital day 9 (06/14/2025). CSF analysis was acellular, without evidence of bacterial growth, HSV, tuberculosis, or JC virus by polymerase chain reaction. CSF HSV-1 and HSV-2 DNA PCR were not detected. CSF EBV DNA PCR was not obtained at the time of lumbar puncture, as tissue biopsy was being concurrently pursued for definitive diagnosis.

Given the multiple hepatic lesions identified on cross-sectional imaging, interventional radiology-guided liver biopsy was selected for tissue diagnosis. The hepatic lesions were more accessible, with significantly lower procedural risk compared to biopsy of an intramedullary spinal cord lesion, which carries substantial risk of worsening neurological deficits. Liver biopsy was performed on 06/16/2025. Histopathological examination demonstrated EBV-positive DLBCL. Given the presence of confirmed lymphomatous disease outside the CNS, the diagnosis was classified as AIDS-associated systemic DLBCL with secondary CNS involvement.

The National Comprehensive Cancer Network (NCCN) guidelines recommend that workup for suspected PCNSL include holding steroids prior to the diagnostic procedure if possible, biopsy with the least invasive approach, and CSF sampling (15-20 mL to increase diagnostic yield) with flow cytometry, cytology, cell count, and polymerase chain reaction of MYD88 [12]. In this case, dexamethasone was administered prior to tissue sampling, which deviates from the NCCN recommendation. However, the acute neurological deterioration with impending spinal cord injury necessitated urgent corticosteroid therapy. Additionally, tissue diagnosis was obtained from the liver rather than the CNS lesion, reducing the potential confounding effect of steroids on tumor histopathology. For HIV-positive patients, ART should be incorporated into treatment, and consultation with an HIV specialist is important to optimize compatibility with systemic therapy [4,13].

ART was initiated during hospitalization with dolutegravir 50 mg by mouth daily and emtricitabine-tenofovir disoproxil fumarate 200 mg-300 mg by mouth daily in coordination with the infectious disease service. Trimethoprim-sulfamethoxazole 800 mg-160 mg by mouth daily was continued for Pneumocystis jirovecii prophylaxis, given the CD4 count of less than 20 cells/μL. Additional supportive medications included ceftriaxone 2 g intravenously daily, nystatin 100,000 units/mL oral suspension 5 mL four times daily for oral candidiasis prophylaxis, pantoprazole 40 mg by mouth twice daily, folic acid 1 mg by mouth daily, enoxaparin 40 mg subcutaneously daily for venous thromboembolism prophylaxis, and a daily multivitamin.

The patient was subsequently transferred in stable condition to University Hospital for initiation of cyclophosphamide, doxorubicin, vincristine, and prednisone (CHOP) chemotherapy.

## Discussion

Importance of maintaining high clinical suspicion

The rapid progression from initial presentation with perianal abscess to profound neurological compromise reinforces the critical need for clinicians to remain vigilant for CNS involvement when managing patients with a new HIV diagnosis, especially in the interval before CD4 count results become available. The initial CD4 count was pending at admission; the subsequent result of 20 cells/μL placed this patient in the highest risk category for opportunistic infections and AIDS-defining malignancies. Risk for CNS non-Hodgkin lymphoma is particularly elevated in those with profound immunosuppression, and declining CD4 counts confer a proportionally greater risk for CNS lymphoma relative to other lymphoma subtypes [7].

Rarity of spinal cord lymphoma

Spinal cord lymphoma, whether primary or secondary, remains exceptionally rare and underreported. The clinical manifestations of PCNSL lack specificity and can encompass fatigue, cognitive decline, headache, and focal neurological deficits. In uncommon cases, disease may be confined to the leptomeningeal compartment or, rarely, to the spinal cord alone [2]. In a Mayo Clinic series of 14 individuals with primary intramedullary spinal cord lymphoma, the majority were initially misdiagnosed with CNS demyelinating disease, and a median of eight months elapsed before the correct diagnosis was reached. Even with chemotherapy, outcomes were poor, with only 36% surviving to two years [3]. A systematic review cataloging primary cauda equina lymphoma found just 18 reported cases, with women diagnosed at a mean age of 61.25 years and men at 50 years. Survival was notably poor, with only 35% of patients remaining alive at 18 months, underscoring the difficult prognosis associated with this entity [4]. The normal brain MRI in this case could have falsely reassured clinicians, had spinal imaging not been pursued.

Diagnostic reclassification

Although the initial clinical presentation - an intramedullary spinal cord mass with no intracranial lesions in a profoundly immunosuppressed patient - was highly suggestive of PCNSL, the identification of multiple hepatic lesions and subsequent liver biopsy confirming EBV-positive DLBCL established systemic disease. This necessitated reclassification from PCNSL to AIDS-associated systemic DLBCL with secondary CNS involvement [2]. The mixed upper and lower motor neuron presentation, combined with multifocal vertebral body lesions and hepatic involvement, was more consistent with systemic DLBCL with secondary CNS involvement than with isolated PCNSL. This distinction carries important therapeutic implications, as treatment must address both the systemic and CNS compartments simultaneously [2].

The decision to pursue liver biopsy rather than spinal cord biopsy for tissue diagnosis was guided by the greater accessibility and lower procedural morbidity of the hepatic lesions compared to biopsy of an intramedullary spinal cord lesion in a patient with progressive myelopathy.

Cauda equina syndrome as a neurosurgical emergency

Cauda equina syndrome represents a true neurosurgical emergency regardless of etiology. The classic presentation includes bilateral lower extremity weakness, urinary retention, and saddle anesthesia. Prompt recognition and intervention are essential to prevent permanent paralysis and loss of bowel and bladder function. In patients with spinal cord compression due to lymphoma, response to treatment - defined as improvement in neurological deficit - approaches 90% when diagnosed and treated early [14].

Differential diagnosis

The differential diagnosis for spinal cord lesions in HIV/AIDS patients is broad and includes primary CNS lymphoma, toxoplasmosis, tuberculosis (Pott’s disease), CMV myelitis, varicella-zoster virus myelitis, progressive multifocal leukoencephalopathy, and metastatic disease from systemic lymphoma. When evaluating CNS mass lesions in patients with AIDS, the leading diagnostic considerations include PCNSL, tuberculosis, and endemic mycoses. Notably, lymphoma and toxoplasmosis can appear identical on imaging, as both characteristically produce ring-enhancing lesions [15]. A summary of the differential diagnosis for spinal cord lesions in HIV/AIDS patients, including characteristic imaging and CSF findings, is presented in Table 2.

**Table 2:** Differential diagnosis of spinal cord lesions in HIV/AIDS patients Source: Content synthesized from published literature [2-4,7-10,15-17]. Abbreviations: CNS, central nervous system; EBV, Epstein-Barr virus; CMV, cytomegalovirus; VZV, varicella-zoster virus; PML, progressive multifocal leukoencephalopathy; NHL, non-Hodgkin lymphoma; PCNSL, primary central nervous system lymphoma; CSF, cerebrospinal fluid; AFB, acid-fast bacilli; PCR, polymerase chain reaction; PET/CT, positron emission tomography/computed tomography

Diagnosis	Etiology	Typical Imaging Findings	CSF Findings	Distinguishing Features
Primary CNS lymphoma	EBV-driven B-cell proliferation in immunosuppressed patients	Enhancing intramedullary or periventricular lesions; may be solitary or multifocal; homogeneous enhancement in HIV	Elevated protein; EBV DNA PCR positive (sensitivity 83%, specificity 95%); cytology/flow cytometry may show monoclonal B cells	Strongest association with profound immunosuppression (CD4 <50); may present without intracranial lesions; tissue biopsy is gold standard
Toxoplasmosis	*Toxoplasma gondii* reactivation	Multiple ring-enhancing lesions with surrounding edema, typically at the basal ganglia and corticomedullary junction; spinal involvement is rare	Nonspecific; Toxoplasma PCR may be positive	Positive Toxoplasma IgG serology; empiric treatment trial with sulfadiazine/pyrimethamine; radiographic improvement within 2-4 weeks distinguishes it from lymphoma
Tuberculosis (Pott's disease)	Mycobacterium tuberculosis	Vertebral body destruction with paraspinal abscess; epidural involvement with cord compression; gibbus deformity in advanced cases	Lymphocytic pleocytosis; elevated protein; low glucose; AFB smear/culture; TB PCR	Vertebral body collapse and paravertebral abscess formation; endemic exposure history; QuantiFERON may be indeterminate in severe immunosuppression
CMV myelitis	Cytomegalovirus reactivation	T2 hyperintensity of the conus medullaris and cauda equina; leptomeningeal enhancement	Polymorphonuclear pleocytosis; CMV DNA PCR positive; elevated protein	Rapidly progressive polyradiculopathy with predominant lower extremity involvement; concurrent CMV retinitis or colitis may be present
VZV myelitis	Varicella-zoster virus reactivation	Longitudinally extensive T2 hyperintensity; dorsal cord predominance	Lymphocytic pleocytosis; VZV DNA PCR positive; VZV IgM/IgG in CSF	Often preceded by dermatomal zoster rash; may present without rash (zoster sine herpete); dorsal column involvement predominates
PML	JC virus reactivation	Multifocal white matter T2/FLAIR hyperintensities without enhancement or mass effect; spinal cord involvement is exceedingly rare	JC virus DNA PCR positive	Predominantly cerebral white matter disease; no mass effect or enhancement; spinal involvement is exceptionally rare
Metastatic systemic lymphoma	Hematogenous or CSF dissemination from systemic NHL	Epidural or leptomeningeal enhancement; vertebral body lesions may be indistinguishable from PCNSL on imaging	Similar to PCNSL; cytology and flow cytometry may show malignant cells	Evidence of systemic lymphoma on staging (PET/CT, bone marrow biopsy); epidural involvement is more common than intramedullary
Spinal epidural abscess	Bacterial (*Staphylococcus aureus* most common)	Epidural fluid collection with peripheral enhancement; associated vertebral osteomyelitis/discitis	Elevated WBC and protein; culture may be positive if ruptured into the subarachnoid space	Fever, localized back pain, and rapidly progressive neurologic deficits; blood cultures often positive; surgical emergency

Role of EBV PCR in diagnosis

EBV PCR from CSF plays an important diagnostic role in AIDS-related PCNSL. A meta-analysis of 12 studies including 141 patients with AIDS-related PCNSL found that CSF EBV-DNA had a pooled sensitivity of 83%, specificity of 95%, and an area under the curve of 0.94, concluding it can be a reliable diagnostic biomarker [16]. Identification of EBV DNA in CSF via PCR testing should heighten suspicion for CNS lymphoma, particularly when quantitative assays reveal concentrations exceeding 10,000 copies/mL [15]. Quantification of EBV DNA can improve specificity for PCNSL compared with qualitative detection (96% vs 66% when a cutoff of 10,000 copies/mL is used) [2].

The gold standard for diagnosis remains tissue biopsy [2]. In the present case, CSF EBV DNA PCR was not obtained at the time of lumbar puncture. While serum EBV DNA was elevated at 2,300 IU/mL, CSF EBV PCR would have provided additional diagnostic value, particularly given its high sensitivity and specificity for CNS lymphoma [16]. For individuals with advanced AIDS who cannot undergo tissue sampling, the presence of a fluorodeoxyglucose (FDG)-avid CNS lesion, coupled with a high CSF EBV viral load, carries a strong positive predictive value and may provide sufficient justification to initiate treatment [17].

Treatment considerations

Given the reclassification of this case as systemic DLBCL with secondary CNS involvement, treatment must address both systemic and CNS disease. The patient was transferred for initiation of CHOP chemotherapy. R-CHOP, the addition of rituximab to CHOP, is the standard first-line regimen for CD20-positive DLBCL and has been shown to significantly improve response rates and overall survival compared to CHOP alone [2]. The histopathology in this case confirmed CD20-positive DLBCL, and the omission of rituximab warrants consideration. The decision to initiate CHOP without rituximab was made by the oncology team at the receiving institution, and the rationale was not available to the authors at the time of this report.

Additionally, CHOP alone has limited penetration across the blood-brain barrier and is insufficient as CNS-directed therapy [2]. For patients with concurrent systemic and CNS lymphoma, incorporation of CNS-penetrating agents such as high-dose methotrexate is recommended [2,13]. Whether intrathecal chemotherapy or high-dose methotrexate was planned following CHOP initiation is not known, as long-term follow-up data from the receiving institution are not yet available.

For HIV-positive patients, ART initiation is essential and should be incorporated as part of the treatment plan [13]. Dolutegravir-based regimens are preferred due to minimal drug-drug interactions with chemotherapy agents [13]. In this case, ART was initiated with dolutegravir and emtricitabine-tenofovir disoproxil fumarate, in coordination with the infectious disease service. Early ART initiation in HIV-associated lymphoma may contribute to immune reconstitution and improve outcomes, though careful coordination with chemotherapy timing is necessary to minimize toxicity [4,13]. Avoidance of zidovudine, cobicistat, and ritonavir is strongly recommended when administering concurrent chemotherapy due to drug-drug interactions, including additive myelosuppression with zidovudine and CYP3A4 inhibition with cobicistat and ritonavir, which can increase chemotherapy toxicity [13]. A summary of antiretroviral medications to avoid during concurrent chemotherapy is presented in Table 3.

**Table 3:** Antiretroviral medications to avoid during concurrent CHOP chemotherapy due to drug-drug interactions Source: [13] Abbreviations: CYP3A4, cytochrome P450 3A4; CHOP, cyclophosphamide, doxorubicin, vincristine, and prednisone

Medication to Avoid	Interaction Concern
Zidovudine	Additive myelosuppression with cyclophosphamide and doxorubicin
Cobicistat	CYP3A4 inhibition increasing vincristine and doxorubicin toxicity
Ritonavir	Strong CYP3A4 inhibition with risk of severe vincristine neurotoxicity

Limitations

This report has several limitations inherent to its design as a single case report, which limits generalizability to broader patient populations. Tissue biopsy of the spinal cord lesion itself was not performed; the diagnosis of lymphoma was confirmed through interventional radiology-guided liver biopsy demonstrating EBV-positive DLBCL, and the spinal involvement was inferred based on characteristic imaging findings and clinical context. CSF EBV DNA PCR was not obtained, which represents a limitation given the central role of this test in the diagnostic workup of CNS lymphoma [16]. CSF analysis was acellular and did not yield a cytological or flow cytometric diagnosis, which may reflect the limited sensitivity of a single lumbar puncture in leptomeningeal disease. Long-term treatment response and outcome data following initiation of CHOP chemotherapy are not yet available, and follow-up imaging to assess treatment response of the spinal lesion has not been reported. Additionally, the indeterminate QuantiFERON-TB result secondary to profound immunosuppression limited the ability to definitively exclude tuberculosis as a contributing etiology. This case was initially approached as suspected PCNSL; however, the identification of systemic disease on liver biopsy necessitated reclassification as systemic DLBCL with secondary CNS involvement, which affects the applicability of PCNSL-specific treatment guidelines discussed herein. Despite these limitations, this case provides valuable clinical insight into an underrecognized presentation of AIDS-associated CNS lymphoma involving the spinal cord.

## Conclusions

This case underscores the importance of maintaining a broad differential diagnosis, including both PCNSL and systemic DLBCL with secondary CNS involvement, in HIV-positive patients presenting with new-onset myelopathy or signs of cauda equina syndrome. Although PCNSL was initially suspected, liver biopsy confirmed systemic EBV-positive DLBCL, highlighting the necessity of comprehensive systemic staging in all patients with suspected CNS lymphoma. Spinal involvement remains underreported in advanced AIDS, and the absence of intracranial disease on brain MRI should not preclude this diagnosis.

Early, comprehensive spinal imaging, even when brain imaging is unremarkable, is essential to enable timely diagnosis, decompressive intervention when indicated, and initiation of definitive therapy. The degree of immunosuppression at presentation and the rapidity of neurological decline highlight the need for clinicians to maintain a low threshold for spinal neuroimaging in any HIV-positive patient with unexplained lower extremity symptoms, bladder dysfunction, or new myelopathy. Multidisciplinary coordination among neurosurgery, oncology, and infectious disease is essential. For systemic DLBCL with secondary CNS involvement, treatment should incorporate rituximab-based systemic immunochemotherapy alongside CNS-directed therapy, with concurrent ART initiation.

## References

[1] Kimani SM, Painschab MS, Horner MJ, Muchengeti M, Fedoriw Y, Shiels MS, Gopal S (2020). Epidemiology of haematological malignancies in people living with HIV. Lancet HIV.

[2] Tan IL, Smith BR, von Geldern G, Mateen FJ, McArthur JC (2012). HIV-associated opportunistic infections of the CNS. Lancet Neurol.

[3] Flanagan EP, O'Neill BP, Porter AB (2011). Primary intramedullary spinal cord lymphoma. Neurology.

[4] Lapolla P, Maiola V, Familiari P (2024). Primary cauda equina lymphoma mimicking meningioma. J Clin Med.

[5] Shah T, Venur VA (2023). Central nervous system lymphoma. Semin Neurol.

[6] Schaff LR, Grommes C (2022). Primary central nervous system lymphoma. Blood.

[7] Hernández-Ramírez RU, Qin L, Lin H (2019). Association of immunosuppression and HIV viraemia with non-Hodgkin lymphoma risk overall and by subtype in people living with HIV in Canada and the USA: a multicentre cohort study. Lancet HIV.

[8] Kawasaki K, Wakabayashi K, Koizumi T, Tanaka R, Takahashi H (2002). Spinal cord involvement of primary central nervous system lymphomas: histopathological examination of 14 autopsy cases. Neuropathology.

[9] Traul DE, Shaffrey ME, Schiff D (2007). spinal-cord neoplasms - intradural neoplasms. Lancet Oncol.

[10] Ferreri AJ (2014). Risk of CNS dissemination in extranodal lymphomas. Lancet Oncol.

[11] Antoine JC, Camdessanché JP (2007). Peripheral nervous system involvement in patients with cancer. Lancet Neurol.

[12] (2025). Central nervous system cancers. https://www.nccn.org/guidelines/guidelines-detail.

[13] (2025). B-cell lymphomas. https://www.nccn.org/guidelines/guidelines-detail?category=1&id=1480.

[14] Aabo K, Walbom-Jørgensen S (1986). Central nervous system complications by malignant lymphomas: radiation schedule and treatment results. Int J Radiat Oncol Biol Phys.

[15] National Institutes of Health, Centers for Disease Control and Prevention, HIV Medicine Association, and Infectious Diseases Society of America (2025). Guidelines for the Prevention and Treatment of Opportunistic Infections in Adults and Adolescents With HIV. Guidelines for the Prevention and Treatment of Opportunistic Infections in Adults and Adolescents With HIV.

[16] Ding X, Liang T, Liang B (2022). Diagnostic value of EBV-DNA in CSF for PCNSL in AIDS patients with focal brain lesions: a meta-analysis of diagnostic test. Medicine (Baltimore).

[17] Yarchoan R, Uldrick TS (2018). HIV-associated cancers and related diseases. N Engl J Med.

